# The Discrepancy Between Influenza Vaccine Recommendation and Uptake Among Healthcare Workers in China: A Multicenter Cross-Sectional Study

**DOI:** 10.3390/vaccines14020166

**Published:** 2026-02-11

**Authors:** Xingxing Zhang, Chenyan Jiang, Lin Sun, Yu Xiong, Qiangling Yin, Ju Wang, Xiao Yu, Qing Duan, Yinzi Chen, Xin You, Shuaixing Wang, Xiaoxu Zeng, Lei Yang, Dayan Wang

**Affiliations:** 1 Chinese National Influenza Center, National Institute for Viral Disease Control and Prevention, Chinese Center for Disease Control and Prevention, Beijing 102026, China; zhangxx@ivdc.chinacdc.cn (X.Z.); wangsx@ivdc.chinacdc.cn (S.W.); zengxx@ivdc.chinacdc.cn (X.Z.); yanglei@ivdc.chinacdc.cn (L.Y.); 2 Shanghai Municipal Center for Disease Control and Prevention (Shanghai Academy of Preventive Medical Sciences), Shanghai 200336, China; jiangchenyan@scdc.sh.cn (C.J.); chenyinzi@scdc.sh.cn (Y.C.); 3 Infectious Disease Prevention and Control Institute of Shandong Center for Disease Control and Prevention/Shandong Institute of Preventive Medicine/Shandong Provincial Key Laboratory of Intelligent Monitoring, Early Warning, and Prevention of Infectious Diseases, Jinan 250014, China; sunli_1825@163.com (L.S.); sdcdcdq@163.com (Q.D.); 4 Chongqing Municipal Center for Disease Control and Prevention (Chongqing Academy of Preventive Medicine), Chongqing 400707, China; xiongyu1139@163.com (Y.X.); chongqingcfswangju@163.com (J.W.); 5 Hubei Provincial Center for Disease Control and Prevention, Wuhan 430079, China; yinql18@163.com (Q.Y.); neverabc@163.com (X.Y.); 6 Chinese Preventive Medicine Association, Beijing 100062, China; youx@cpma.org.cn

**Keywords:** healthcare workers, influenza vaccination, knowledge, attitude, practice (KAP), recommendation-uptake discrepancy, China

## Abstract

**Background:** Healthcare workers (HCWs) are pivotal in influenza containment, serving as both high-risk individuals and vaccine advocates. However, influenza vaccination coverage among Chinese HCWs remains suboptimal. Existing research is often constrained by limited geographic representativeness or non-robust designs. This study provides a robust, nationwide assessment of influenza vaccine uptake and recommendation behaviors among HCWs in China. **Methods:** A multicenter cross-sectional survey was conducted in late 2025 across four Chinese provinces (Shanghai, Shandong, Chongqing, and Hubei). A total of 390 frontline HCWs—only those defined as directly engaged in influenza management and prevention—from 48 hospitals (primary, secondary, and tertiary levels) completed validated electronic questionnaires. A multinomial logistic regression model was employed to identify determinants of personal vaccine uptake behavior among HCWs. **Results:** Overall influenza knowledge was moderate, with notable gaps in recognizing typical symptoms (29.23%), southern China’s peak season (31.03%), and optimal vaccination timing (55.38%). A striking “recommendation-uptake disparity” was observed: while 93.6% of HCWs recommended the vaccine to patients, only 22.3% received it annually themselves. A multinomial regression revealed that being a nurse (vs. doctor: OR = 3.11, 95% CI: 1.28–7.53) or female (vs. male: OR = 3.08, 95% CI: 1.28–7.44) was positively associated with annual vaccination, whereas clinical technicians (vs. doctors: OR = 0.18, 95% CI: 0.03–0.94) showed lower odds. Primary barriers to personal vaccination included inconvenience (49.5%), perceived high cost (16.2%), and efficacy concerns (19.5%). **Conclusions:** This study highlights a significant gap between high recommendation rates and low personal uptake among HCWs in China. The findings underscore the need for multifaceted interventions, including workplace-based reminder systems, free vaccination policies, and tailored education, to optimize coverage and strengthen the role of HCWs in national influenza prevention.

## 1. Introduction

Influenza is a seasonal acute respiratory infection that imposes a significant global disease burden [1,2]. According to the World Health Organization (WHO) [3], it causes approximately 1 billion cases annually worldwide, with 3 to 5 million resulting in severe illness. Seasonal influenza epidemics are also responsible for an estimated 290,000 to 650,000 respiratory-related deaths each year.

As the frontline of influenza management, healthcare workers (HCWs) play a pivotal role in disease control through their knowledge, attitudes, and practices (KAP). Prioritizing HCW vaccination is essential not only to mitigate their high occupational exposure and prevent nosocomial transmission—a strategy recommended by the WHO and implemented in over 40 regions, including China [4,5]—but also to leverage their professional influence. By virtue of their expertise and the trust they command, HCWs serve as critical community advocates whose recommendations significantly enhance public confidence and overall vaccine coverage.

Influenza vaccination coverage among Chinese HCWs remains suboptimal [6]. Specific vaccination rates vary significantly depending on the study region, methodology, and timeframe [6,7,8,9,10]. Nevertheless, existing research on influenza vaccination and recommendation among Chinese healthcare workers (HCWs) faces three primary constraints: (1) a lack of nationwide representativeness, with most studies restricted to specific regions [9,10]; (2) methodological limitations, characterized by a reliance on convenience online sampling [6,7] rather than rigorous stratified inclusion; and (3) the scarcity of research on dual HCW vaccination and recommendation. To bridge these gaps, this study utilizes the robust surveillance network of the Chinese National Influenza Center (CNIC). By conducting a multicenter cross-sectional survey across four provinces with diverse economic and geographic profiles, this study offers enhanced design robustness and a more comprehensive reflection of the national landscape.

Specifically, this survey aims to evaluate influenza-related KAP among Chinese HCWs, with a primary focus on identifying the determinants of their vaccine uptake and recommendation behaviors. The resulting evidence base will provide updated scientific insights into HCWs’ perspectives in the post-pandemic era. Ultimately, these findings will serve as a critical reference for optimizing national immunization strategies and bolstering influenza vaccine coverage across the general population.

## 2. Materials and Methods

### 2.1. Settings and Participants

Based on considerations of regional disparities in influenza disease burden, influenza vaccine policy, and socioeconomic development, 4 provinces/municipalities were selected as study sites: Shanghai (East China), Shandong (North China), Chongqing (Southwest China), and Hubei (Central China). It is worth noting that none of these provinces currently has a free influenza vaccination policy targeting HCWs in place.

The provincial centers for disease control and prevention (CDC) coordinated the survey in each site. In each of the 4 provinces, 4 cities or districts were selected for inclusion. Within each city/district, one tertiary, one secondary, and one primary hospital were recruited, resulting in a total of 48 hospitals participating in this study. A minimum of 8 HCWs were recruited into the study from each hospital upon obtaining informed consent. Here, HCWs specifically refers to frontline personnel directly tasked with influenza prevention and control, ranging from clinical diagnosis to vaccination and public health education. In total, 390 HCWs were targeted for survey participation across the 4 settings.

### 2.2. Questionnaire Survey

The validated electronic questionnaire was administered via QR codes or web links provided by local CDC personnel during face-to-face recruitment from September to October 2025. This timeline ensured that data collection was completed prior to the peak influenza season, thereby mitigating the potential influence of an active epidemic on participants’ KAP.

The questionnaire was developed specifically for this study based on a comprehensive review of the literature and multiple rounds of expert review. Compared with previous similar questionnaires, this survey features the following highlights: 1. It simultaneously examines the KAP of HCWs from the dual perspectives of being both patients and healthcare providers. 2. It concurrently focuses on HCWs’ behaviors regarding both influenza vaccination and recommendation, as well as their respective influencing factors.

Additionally, the questionnaire, which consisted of 47 items, was designed to be completed in approximately 8~10 min. The structure and content of the questionnaire were as follows: Section 1: Sociodemographic Characteristics. This section collected background information, including, but not limited to, age, gender, and educational background. Section 2: Influenza-Related Knowledge and Attitudes. This part assessed participants’ knowledge and perceptions of influenza viruses and vaccines. Section 3: Influenza-Related Behaviors. This section evaluated self-reported practices in two key domains: (a) diagnosis and treatment, and (b) preventive measures and vaccination. Section 4: Health Communication Practices. This part evaluated two aspects: (a) participants’ exposure to health education campaigns, and (b) their own delivery of science popularization activities within the community. Section 5: Recommendations for Influenza Control. This final section focused on the challenges they encountered in influenza clinical and prevention management, as well as their concrete suggestions for public health strategy optimization.

### 2.3. Data Analysis

#### 2.3.1. Descriptive Analysis

Firstly, the demographic characteristics of the study participants were stratified and summarized in the form of counts and percentages, which consisted of province/municipalities, hospital level, position, work experience (years), gender, age group (years), ethnicity, marriage, health status, education background, and monthly income per capita (yuan). Secondly, correct rates of 13 questions on influenza knowledge and beliefs were calculated among all the participants and by province, respectively. Based on the scoring rule (10 points for correct, 0 for incorrect), a score for each of the 13 items was calculated for every participant, and the total score was subsequently computed. Then, the score group distribution was plotted among all the participants and by provinces, respectively. Thirdly, behaviors on influenza diagnosis, testing, treatment, vaccination, and health education, et.al., were also analyzed in terms of frequency and percentages among all and by province, respectively.

#### 2.3.2. Logistic Regression Analysis

Among all behaviors, “whether to get vaccinated against flu annually” was the focus of the influencing factors analysis.

In univariable analysis, we regressed each potential influencing factor against the above behavior. Based on the previous literature and established knowledge, the factors included the demographic characteristics of the participants and their knowledge and attitude towards flu. More details on the univariate analysis of the vaccination behavior can be found in the Supplementary Materials (Table S1).

In multivariable analysis, all the above potential factors were considered for inclusion in logistic analysis. The theoretical foundation has two highlights: 1. This study is an observational study, and it is usually appropriate to directly conduct multivariate analysis to fully control for confounding factors and simultaneously reduce Type I errors; 2. The sample size of this study is relatively adequate, with a limited number of independent variables (*N* = 390, 10 categorical variables), so conducting multivariate analysis directly can help retain all potential influencing factors as much as possible. Then, odds ratios (ORs) and their 95% confidence intervals (CIs) extracted from the multivariable regression model were used to measure the magnitude of the influence of a factor on the behavior. It is worth noting that in the multivariable logistic regression for “whether to get vaccinated against flu,” since the dependent variable has three levels—“vaccinated annually,” “vaccinated but not annually,” and “never vaccinated”—this study employed a multinomial logistic regression analysis. Detailed data of the multivariate analysis of the vaccination behavior can be found in the Supplementary Materials (Table S2).

All the above analyses were conducted using R version 4.4.2 (2024-10-31).

## 3. Results

### 3.1. Demographic Characteristics

A total of 390 HCWs were included in this survey. The distribution across provinces was relatively balanced: 97 (24.87%) from Shanghai, 96 (24.62%) from Shandong, 101 (25.90%) from Hubei, and 96 (24.62%) from Chongqing. In terms of hospital level, participants were evenly distributed among primary, secondary, and tertiary institutions, with 128 (32.82%), 124 (31.79%), and 138 (35.38%) from each level, respectively. Among them, physicians constituted the largest group (228, 39.65%), followed by nurses (139, 48.35%) and allied clinical technicians (23, 12.00%). Regarding work experience, slightly less than half (177, 42.04%) had worked for 10–20 years, while 93 (11.05%) had less than 10 years, 85 (30.29%) had 20–30 years, and 35 (16.63%) had over 30 years of experience. Additional sociodemographic details are presented in Table 1.

### 3.2. Knowledge and Attitudes Regarding Influenza and Its Vaccines

**Figure 1:**
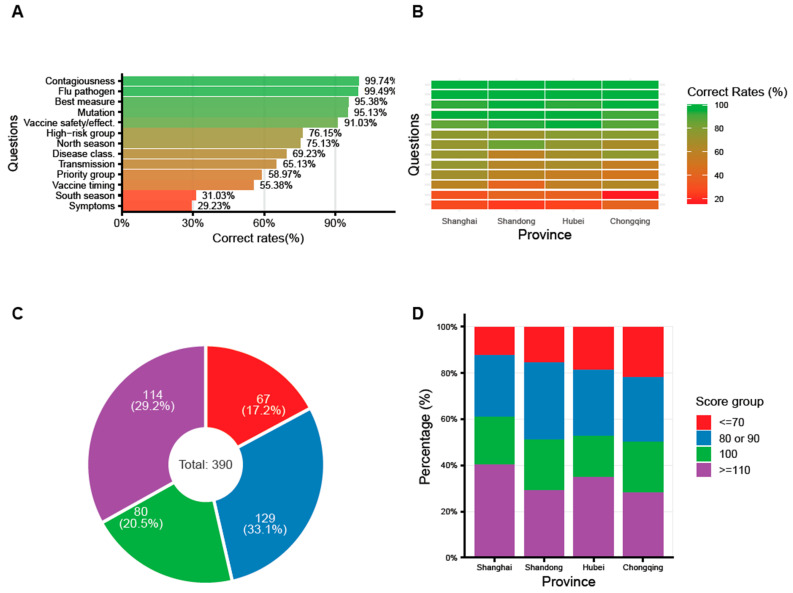
**Knowledge and attitude of influenza among 390 HCWs in 4 provinces of China, 2025**. (**A**). Correct rates of flu-associated knowledge questions among all the participants, (**B**). Correct rates among participants in 4 provinces/municipalities, respectively, (**C**). Score group among all participants if 10 points for correct, 0 for incorrect, (**D**). Score group distribution in 4 provinces/municipalities, respectively.

The questionnaire included 13 items related to influenza knowledge and attitudes. The three questions with the highest correct rates were: “Is influenza highly contagious or not” (99.74%), “What’s the pathogen causing flu” (99.49%), and “What’s the most effective measure to prevent flu” (95.38%). The three questions with the lowest correct response rates were: “What are the typical symptoms of influenza” (29.23%), “The peak season for influenza in southern China” (31.03%), and “The recommended timing for influenza vaccination” (55.38%) (Figure 1A). The overall scores for the above questions were categorized into the following ranges: ≤70, 80–90, 100, and ≥110 (Figure 1C). The highest score group (≥110) had the largest number of participants, with 129 individuals (33.1%). The second largest group was the 80–90 score range, comprising 114 participants (29.2%). Those scoring 100 points accounted for 80 participants (20.5%), while the lowest scoring group (≤70) included 67 participants (17.2%). Further details on correct rates and score distribution by province are provided in Figure 1B,D.

### 3.3. Influenza-Related Behaviors: The Recommendation-Uptake Disparity

In the context of influenza-related practices, information under two different scenarios was investigated: one when HCWs went to see a doctor themselves (Table 2, Scenario A), and the other when they treated patients (Table 3, Scenario B).

In scenario A, most HCWs have contracted influenza (235, 60.3%), with nearly half having experienced it in the previous year (190, 48.7%). Among those infected last year, nearly half self-diagnosed based on personal experience (113, 49.8%), while less than one-third were confirmed by a positive influenza test at a hospital (61, 26.9%). The majority of those infected took oral medication for treatment (196, 86.3%). Regarding influenza vaccination, less than one-fourth of individuals received the vaccine annually (87, 22.3%), while more than half ever got vaccination but not every year (213, 54.6%). Another one-fourth never got the flu shot (90, 23.1%). Only one-fourth were vaccinated against influenza last year (105, 26.9%). The reasons for not receiving the influenza vaccine vary considerably. Approximately half cited “forgetting to get vaccinated” (51, 56.7%), a quarter considered it “too expensive” (21, 23.3%), while some others mentioned that “most relatives or friends didn’t get vaccinated” (15, 16.7%) or believed the “vaccine is ineffective” (13, 14.4%). Over sixty percent (253, 64.9%) of HCWs reported that when they visited a doctor for ILI, the physician would recommend the influenza vaccine to them. As patients, the greatest challenge they faced regarding influenza vaccination was “the inconvenience of annual vaccination” (193, 49.5%), followed by “uncertain effectiveness” (76, 19.5%). Other associated details are presented in Table 2.

In scenario B, when treating patients suspected of contracting influenza, the majority of doctors would order influenza testing (223, 97.8%). Among these, less than half would order both antigen and nucleic acid tests (97, 43.5%), over one-third would order only antibody tests (85, 38.1%), and less than one-fifth would order only nucleic acid tests (41, 18.4%). When treating influenza cases, most doctors inquired about the illness status of family members (226, 99.1%) or classmates (for students) (225, 98.7%) and provided protective advice. Most physicians (365, 93.6%) recommend the influenza vaccine to their patients, primarily because they believe “the vaccine is safe and effective.” (352, 96.4%). Among the very few HCWs who do not recommend the influenza vaccine, the reasons include believing “it is too expensive” (6, 24.0%), “ it is unsafe” (3, 12.0%), “it is ineffective” (7, 28.0%), and “most relatives and friends didn’t get vaccinated“ (8, 32.0%). When HCWs recommended the influenza vaccine to patients in clinical practice, over 60% of patients expressed “interest and willingness to get vaccinated” (232, 63.6%). A smaller portion adopted a “neutral attitude, expressing indifference” (105, 28.8%), while very few stated they are “not interested and deem it unnecessary” (28, 7.7%).

### 3.4. Predictors of Influenza Vaccine Uptake

**Figure 2:**
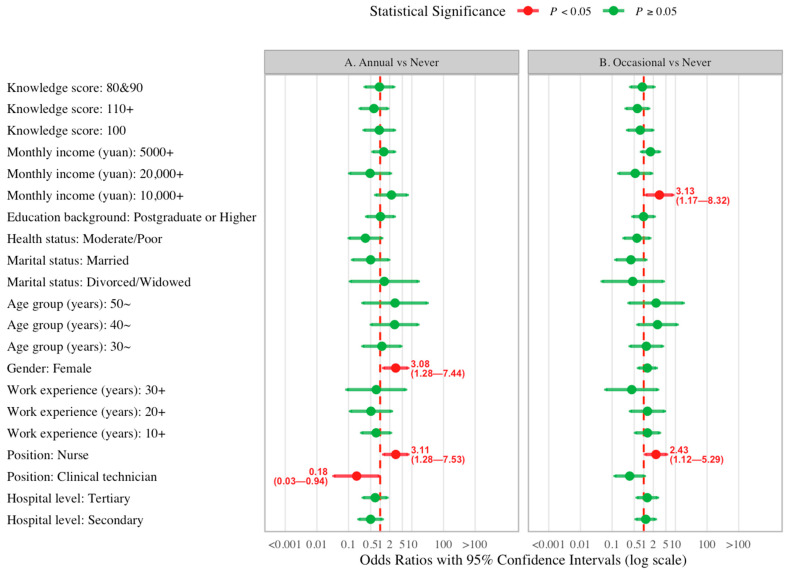
Multivariate logistic regression on behavior “whether to get vaccinated against flu annually” among the 390 HCWS in 4 provinces of China, 2025. A. Annually vaccinated vs. never vaccinated, B. Occasionally vaccinated vs. never vaccinated. Reference group (Ref.) of Hospital level: Primary, Ref. of Position: Doctor, Ref. of Work experience (years): <10, Ref. of Gender: Male, Ref. of Age group (years): 20–29, Ref. of Marital status: Single, Ref. of Health status: Healthy, Ref. of Education Background: College or below, Ref. of Monthly income (yuan): <5000, Ref. of Knowledge score: ≤70.

Figure 2 presents a forest plot summarizing the results of the multinomial logistic regression analysis examining factors associated with influenza vaccination behavior among the HCWs. The analysis compared two categories: (A) “Annual vaccination” versus “Never vaccinated,” and (B) “Occasional vaccination” (vaccinated but not annually) versus “Never vaccinated.” In comparison A, two factors were significantly associated with higher odds of annual vaccination relative to never being vaccinated. As shown in the figure, female vs. male (OR 3.08, 95%CI 1.28–7.44), nurse vs. doctor (OR 3.11, 95%CI 1.28–7.53) were associated with increased likelihood of annual vaccination. However, clinical technician vs. doctor (OR 0.18, 95%CI 0.03–0.94) was related to a lower possibility of getting vaccinated annually. In comparison B, two factors were also significantly associated with higher odds of occasional vaccination relative to never being immunized. Nurse vs. doctor (OR 2.43, 95% CI 1.12–5.29) and higher monthly income per capita (e.g., 10,000+ yuan: OR 3.13, 95% CI 1.17–8.32) were associated with a higher possibility of occasional vaccination. More information on the univariate or multivariate logistic analyses of “whether to get vaccinated against flu” was detailed in Supplementary Material (Tables S1 and S2).

## 4. Discussion

This multicenter survey provides an overview of the KAP regarding influenza among 390 HCWs in four diverse provinces of China, with a specific focus on vaccination behaviors. Our study reveals several important findings, some of which were unexpected:

Firstly, the results revealed an uneven knowledge base among the surveyed HCWs. In terms of the correct rates, while HCWs demonstrated excellent understanding of influenza’s etiology and epidemiology, knowledge gaps persisted regarding typical symptoms (29.23%), southern China’s peak season (31.03%), and optimal vaccination timing (55.38%); this discrepancy mirrors findings from the previous studies [9,10]. The lack of awareness regarding local seasonality or vaccination timing is concerning, as it may hinder the timely administration of vaccines during the epidemic season. From another perspective, the knowledge base of HCWs exhibited significant inter-provincial disparities, suggesting that health education should be continuously strengthened, particularly in regions with lower proficiency. Therefore, future education initiatives should transition from general theoretical reviews toward targeted training modules that emphasize actionable clinical details, notably localized seasonality and optimal vaccination windows. Such specialized strategies are essential to narrow regional knowledge gaps and empower HCWs across all provinces to provide precise, time-sensitive guidance to the public.

Secondly, the results demonstrated that personal vaccine uptake among the surveyed HCWs was suboptimal, which has been revealed in previous studies in different regions of China [11,12]. According to self-reported data from the study, only 22.3% of HCWs received the influenza vaccine annually, with a mere 26.9% vaccinated during the 2024–2025 season. This coverage rate falls far below the levels typically observed in Western countries. For instance, 75.9% of HCWs reported receiving influenza vaccination during the 2022–2023 season in the USA [13]. For another example, according to reports from the UKHSA, the influenza vaccination uptake rates among frontline HCWs in the UK were 37.9% and 42.8% for the 2024–2025 and 2023–2024 seasons, respectively [14]. The WHO recommended that health workers should be the target groups of influenza vaccination [15], which also been incorporated into China’s Influenza Vaccination Technical Guidelines [5]. However, real barriers in China exist. In our study, the primary self-reported one was “forgetting to vaccinate,” followed by “perceived high cost”, “low vaccine uptake in social circle”, and “doubts about vaccine efficacy” among those who never get vaccinated. Given that “forgetting” is the primary barrier among those never vaccinated, a qualitative study is warranted to explore the personal health practices and their determinants among HCWs. Regarding the foremost barrier among all the HCWs, “the inconvenience of annual vaccination (49.5%)” topped, suggesting an urgent need for a comprehensive influenza vaccine promotion strategy.

Thirdly, the study identified a high level of willingness among HCWs to recommend the vaccine to their patients. A strong majority (93.6%) recommended vaccination to patients, creating a disparity with their own low uptake rate—a key finding of our study. This paradox may stem from several factors: (1) a role-perception gap, wherein HCWs act as public health advocates rather than as at-risk individuals [16]; (2) structural barriers such as lack of convenient, free access at the workplace [17] or lack of a universal influenza vaccine, which echoes the self-reported barriers to influenza vaccination among HCWs mentioned above; and (3) the persistence of cognitive biases, notably optimism bias, results in the underestimation of personal risk, even among those with professional knowledge, such as the HCWs in the study [18]. Consequently, a multifaceted approach is urgently warranted to bridge the “gap”.

Fourthly, vaccination behavior was shaped by multiple influencing factors. Personal vaccination was significantly associated with occupation (nurses > doctors), gender (female > male), education, income, and health status. Interestingly, the finding that nurses exhibited higher uptake rates than doctors contrasts with typical trends in Western settings [19] but aligns with recent studies conducted in Shanghai and other Chinese regions [9]. This inversion may reflect the higher compliance of nursing staff with hospital infection control directives. Moreover, it suggests that nurses may possess a heightened awareness of their occupational exposure risk to influenza compared to their physician counterparts. Furthermore, the willingness to recommend vaccines was strongly predicted by higher knowledge scores and personal vaccination behavior but was notably lower among clinical technicians compared to physicians. This disparity likely stems from the variation in professional training; while doctors routinely integrate vaccine advocacy into clinical practice, technicians may perceive vaccination guidance as outside their scope of practice or lack specific training on vaccine safety, a barrier also observed in other allied health staff [20].

Taken together, our findings provide some implications for public health policy and practice. It is crucial to implement comprehensive measures to overcome potential barriers and increase influenza vaccination rates among HCWs, which will, in turn, reciprocally enhance their willingness to recommend the influenza vaccine to patients. Specific implications could include the following: (1) Roll out the reminder/reward systems and provide convenient access at the workplace, targeted at HCWs during the vaccination season, to prevent missing shots. For example, during flu seasons, governments or healthcare institutions could provide appropriate rewards to HCWS to promote flu vaccinations [21,22,23,24,25]. Another example, the presence of prominent reminder signs or on-site vaccination stations within the hospital will help HCWS remember to get vaccinated and allow them to do so conveniently, even amidst their busy schedules. (2) Continuing research and publicizing data on vaccine protective effectiveness are warranted among HCWs. Extensive research employing psychological and behavioral theories to explore ways of increasing influenza vaccination rates among HCWs has found that “attitudes toward the safety and efficacy of the influenza vaccine” serve as a significant behavioral predictor [26,27,28]. This suggests that enhancing healthcare workers’ confidence in vaccine effectiveness and addressing vaccine hesitancy are crucial measures for promoting vaccination and improving coverage rates. (3) Continuous efforts should be made to incorporate influenza vaccines into the national immunization program. Concurrently, advocating for the adoption of free vaccination policies across provinces will help further reduce costs and alleviate the “high cost” barrier perceived by HCWs. Some Western countries have their own local strategies for free influenza vaccination, which largely provide policy support for healthcare workers to receive the flu vaccine. For instance, the United Kingdom offers free flu vaccinations through the National Health Service (NHS) to specific eligible groups, including HCWs [29]. The United States does not have a universal free flu vaccine policy at the national level [30,31]. Vaccinations are typically covered by most health insurance plans, while state and local programs offer free or low-cost options for uninsured or underinsured adults [32]. (4) Increasing investment in research and development of a universal influenza vaccine [33], thereby fundamentally streamlining the vaccination process.

This study has limitations inherent to its cross-sectional design, which weakens the strength of causal inference, offering only clues or associations regarding factors influencing influenza vaccination uptake or recommendation. Secondly, self-reported data in the survey may be subject to social desirability, which would introduce information bias. Thirdly, although four provinces were included, the socioeconomic and epidemiological heterogeneity across China’s provinces/municipalities/regions may limit the generalizability of our findings. Expanding surveys to include more provinces is crucial for a national overview. Additionally, longitudinal or interventional designs, which aim to explore the effectiveness of specific measures to increase influenza vaccine coverage, were warranted.

## 5. Conclusions

This study unmasks a striking “recommendation-uptake disparity” among Chinese HCWs. We urgently advocate for comprehensive interventions, specifically the integration of workplace reminder/vaccination systems, free policies, and confidence-building to strengthen the role of HCWs as both protected caregivers and effective advocates in China’s influenza prevention efforts.

## Figures and Tables

**Table 1:** **Demographic characteristics of the 390 HCWs in 4 provinces of China, 2025.**

		n	%
**Province/municipalities**			
	Shanghai	97	24.87%
	Shandong	96	24.62%
	Hubei	101	25.90%
	Chongqing	96	24.62%
**Hospital level**			
	Primary	128	32.82%
	Secondary	124	31.79%
	Tertiary	138	35.38%
**Position**			
	Doctor	228	58.46%
	Nurse	139	35.64%
	Clinical technician *	23	5.90%
**Work experience (years)**			
	0~	93	23.85%
	10~	177	45.38%
	20~	85	21.79%
	30~	35	8.97%
**Gender**			
	Male	102	26.15%
	Female	288	73.85%
**Age group (years)**			
	20~	37	9.49%
	30~	148	37.95%
	40~	156	40.00%
	50~	49	12.56%
**Ethnicity**			
	Han Chinese	380	97.44%
	Other ethnic groups	10	2.56%
**Marital status**			
	Single	45	11.54%
	Married	334	85.64%
	Divorced	10	2.56%
	Widowed	1	0.26%
**Health status**			
	Good	351	90.00%
	Moderate	38	9.74%
	Poor	1	0.26%
**Education background**			
	High School/Vocational School	4	1.03%
	College/University	301	77.18%
	Postgraduate or higher	85	21.79%
**Monthly income per capita (yuan)**			
	0~	134	34.36%
	5000~	175	44.87%
	10,000~	61	15.64%
	20,000~	20	5.13%
**Total**		390	100%

* Note: Clinical technicians referred to healthcare workers who were engaged in specimen testing or pulmonary imaging regarding influenza diagnosis, including laboratory technicians and radiology technicians.

**Table 2:** **Behaviors on influenza diagnosis, testing, treatment, vaccination, and health education among 390 HCWS in 4 provinces/municipalities in China, 2025. (Scenario A: When they saw a doctor).**

		Shanghai *n* (%)	Shandong *n* (%)	Hubei *n* (%)	Chongqing *n* (%)	Total *N* (%)	*p*
**Have you ever contracted influenza?**							<0.05
	Yes	63 (64.95)	54 (56.25)	72 (71.29)	46 (47.92)	235 (60.3%)	
	No	34 (35.05)	42 (43.75)	29 (28.71)	50 (52.08)	155 (39.7%)	
**How many times have you contracted influenza in the past year?**							<0.05
	1	53 (84.13)	44 (81.48)	48 (66.67)	45 (97.83)	190 (48.7%)	
	2	9 (14.29)	7 (12.96)	14 (19.44)	1 (2.17)	31 (7.9%)	
	3	1 (1.59)	3 (5.56)	2 (2.78)	0 (0.00)	6 (1.5%)	
	0	0 (0.00)	0 (0.00)	8 (11.11)	0 (0.00)	8 (2.1%)	
**How did you ascertain that you had influenza in the past year?**							<0.05
	Personal experience	27 (42.86)	27 (50.00)	38 (59.38)	21 (45.65)	113 (49.8%)	
	Clinically diagnosed by a physician	4 (6.35)	14 (25.93)	12 (18.75)	8 (17.39)	38 (16.7%)	
	Tested positive for influenza at a hospital	23 (36.51)	10 (18.52)	13 (20.31)	15 (32.61)	61 (26.9%)	
	Tested positive using a self-purchased influenza test kit	9 (14.29)	3 (5.56)	1 (1.56)	2 (4.35)	15 (6.6%)	
**Time interval between symptoms onset and healthcare seeking**							0.553
	Immediately	25 (39.68)	20 (37.04)	25 (39.06)	16 (34.78)	88 (22.6%)	
	After 1–2 days	19 (30.16)	26 (48.15)	20 (31.25)	16 (34.78)	86 (22.1%)	
	After 3 days to 1 week	6 (9.52)	3 (5.56)	6 (9.38)	3 (6.52)	19 (4.9%)	
	Did not seek	13 (20.63)	5 (9.26)	13 (20.31)	11 (23.91)	42 (10.8%)	
**In the past year, after being diagnosed with influenza, did you take oral antiviral drugs?**							0.079
	Yes	49 (77.78)	49 (90.74)	55 (85.94)	43 (93.48)	196 (86.3%)	
	No	14 (22.22)	5 (9.26)	9 (14.06)	3 (6.52)	31 (13.7%)	
**Do you get a flu shot every year?**							<0.05
	Yes, every year	16 (16.49)	38 (39.58)	10 (9.90)	23 (23.96)	87 (22.3%)	
	Occasionally, but not every year	58 (59.79)	44 (45.83)	70 (69.31)	41 (42.71)	213 (54.6%)	
	Never	23 (23.71)	14 (14.58)	21 (20.79)	32 (33.33)	90 (23.1%)	
**Did you get a flu shot in the past year?**							<0.05
	Yes	19 (19.59)	35 (36.46)	17 (16.83)	34 (35.42)	105 (26.9%)	
	No	78 (80.41)	61 (63.54)	84 (83.17)	62 (64.58)	285 (73.1%)	
**Why didn’t you get a flu shot? ^#^**							
**The price is too expensive**							0.26
	Yes	5 (21.74)	6 (42.86)	3 (14.29)	7 (21.88)	21 (23.3%)	
	No	18 (78.26)	8 (57.14)	18 (85.71)	25 (78.12)	69 (76.7%)	
**It is unsafe**							0.438
	Yes	2 (8.7)	1 (7.14)	1 (4.76)	0 (0.00)	4 (4.4%)	
	No	21 (91.3)	13 (92.86)	20 (95.24)	32 (100.00)	86 (95.6%)	
**It is ineffective**							0.127
	Yes	3 (13.04)	0 (0.00)	2 (9.52)	8 (25.00)	13 (14.4%)	
	No	20 (86.96)	14 (100.00)	19 (90.48)	24 (75.00)	86 (95.6%)	
**Most relatives and friends didn’t get vaccinated**							0.606
	Yes	3 (13.04)	3 (21.43)	2 (9.52)	7 (21.88)	15 (16.7%)	
	No	20 (86.96)	11 (78.57)	19 (90.48)	25 (78.12)	75 (83.3%)	
**The vaccination site was too far, or transportation was inconvenient**							0.147
	Yes	5 (21.74)	0 (0.00)	3 (14.29)	2 (6.25)	10 (11.1%)	
	No	18 (78.26)	14 (100.00)	18 (85.71)	30 (93.75)	80 (88.9%)	
**Didn’t know when or where to get vaccinated**							0.825
	Yes	1 (4.35)	0 (0.00)	1 (4.76)	2 (6.25)	4 (4.4%)	
	No	22 (95.65)	14 (100.00)	20 (95.24)	30 (93.75)	86 (95.6%)	
**Experienced adverse reactions**							0.517
	Yes	3 (13.04)	1 (7.14)	1 (4.76)	1 (3.12)	6 (6.7%)	
	No	20 (86.96)	13 (92.86)	20 (95.24)	31 (96.88)	84 (93.3%)	
**Forgot to get vaccinated**							0.085
	Yes	8 (34.78)	8 (57.14)	13 (61.9)	22 (68.75)	51 (56.7%)	
	No	15 (65.22)	6 (42.86)	8 (38.1)	10 (31.25)	39 (43.3%)	
When you visited the hospital because of ILI *, did the doctor recommend the flu vaccine to you?							0.362
	Yes	56 (57.73)	63 (65.62)	70 (69.31)	64 (66.67)	253 (64.9%)	
	No	41 (42.47)	33 (34.33)	31 (30.69)	32 (33.33)	137 (35.1%)	
**What is the biggest difficulty or concern you or your family/friends have encountered during the influenza vaccination process**							<0.05
	High cost	5 (5.15)	30 (31.25)	10 (9.90)	18 (18.75)	63 (16.2%)	
	Uncertain effectiveness	24 (24.74)	15 (15.62)	18 (17.82)	19 (19.79)	76 (19.5%)	
	Inconvenient location/transportation	12 (12.37)	5 (5.21)	14 (13.86)	9 (9.38)	40 (10.3%)	
	Inconvenience of annual vaccination	51 (52.58)	43 (44.79)	54 (53.47)	45 (46.88)	193 (49.5%)	
	Other	5 (5.15)	3 (3.12)	5 (4.95)	5 (5.21)	18 (4.6%)	

Note: ^#^ This item is a multi-choice question. * ILI is short for influenza-like illness, referring to symptoms “a fever of ≥38 °C, accompanied by either cough or sore throat”, which is a key definition in the influenza surveillance system.

**Table 3:** **Behaviors on influenza diagnosis, testing, treatment, vaccination, and health education among 390 HCWS in 4 provinces/municipalities in China, 2025. (Scenario B: When they treated patients).**

		Shanghai *n* (%)	Shandong *n* (%)	Hubei *n* (%)	Chongqing, *n* (%)	Total *N* (%)	*p*
**Do you order influenza testing if influenza is suspected?**							
	Yes	53 (100.00)	66 (95.65)	56 (98.25)	48 (97.96)	223 (97.8%)	0.434
	No	0 (0.00)	3 (4.35)	1 (1.75)	1 (2.04)	5 (2.2%)	
**Which specific influenza test do you always order?**							
	Antigen testing	29 (54.72)	20 (30.30)	15 (26.79)	21 (43.75)	85 (38.1%)	<0.05
	Nucleic acid testing (e.g., PCR)	10 (18.87)	14 (21.21)	14 (25.00)	3 (6.25)	41 (18.4%)	
	Both the above	14 (26.42)	32 (48.48)	27 (48.21)	24 (50.00)	97 (43.5%)	
**For confirmed influenza cases, do you ask about household symptoms or advise on home protection?**							0.199
	Yes	53 (100.00)	67 (97.10)	57 (100.00)	49 (100.00)	226 (99.1%)	
	No	0 (0.00)	2 (2.9)	0 (0.00)	0 (0.00)	2 (0.9%)	
**For confirmed flu cases in school, do you inquire about classmates’ cases or advise prevention?**							0.072
	Yes	53 (100.00)	66 (95.65)	57 (100.00)	49 (100.00)	225 (98.7%)	
	No	0 (0.00)	3 (4.35)	0 (0.00)	0 (0.01)	3 (1.3%)	
**Would you recommend the influenza vaccine to your patients?**							0.216
	Yes	87 (89.69)	91 (94.79)	94 (93.07)	93 (96.88)	365 (93.6%)	
	No	10 (10.31)	5 (5.21)	7 (6.93)	3 (3.12)	25 (6.4%)	
**Why do you recommend the flu vaccine?**							
**Free vaccination**							<0.05
	Yes	19 (21.84)	31 (34.07)	17 (18.09)	4 (4.3)	71 (19.5%)	
	No	68 (78.16)	60 (65.93)	77 (81.91)	89 (95.7)	294 (80.5%)	
**It’s safe and effective**							0.246
	Yes	84 (96.55)	85 (93.41)	91 (96.81)	92 (98.92)	352 (96.4%)	
	No	3 (3.45)	6 (6.59)	3 (3.19)	1 (1.08)	13 (3.6%)	
**Hospital requires recommendation**							0.538
	Yes	12 (13.79)	7 (7.69)	9 (9.57)	8 (8.6)	36 (9.9%)	
	No	75 (86.21)	84 (92.31)	85 (90.43)	85 (91.4)	329 (90.1%)	
**Personally, will get vaccinated**							<0.05
	Yes	18 (20.69)	12 (13.19)	24 (25.53)	28 (30.11)	82 (22.5%)	
	No	69 (79.31)	79 (86.81)	70 (74.47)	65 (69.89)	283 (77.5%)	
**Why didn’t you recommend the flu vaccine? ^#^**							
**The price is too expensive**							<0.05
	Yes	0 (0.00)	3 (60.00)	3 (42.86)	0 (0.00)	6 (24.0%)	
	No	10 (100.00)	2 (40.00)	4 (57.14)	3 (100.00)	19 (76.0%)	
**It is unsafe**							0.518
	Yes	2 (20.00)	1 (20.00)	0 (0.00)	0 (0.00)	3 (12.0%)	
	No	8 (80.00)	4 (80.00)	7 (100.00)	3 (100.00)	22 (88.0%)	
**It is ineffective**							0.242
	Yes	1 (10.00)	2 (40.00)	2 (28.57)	2 (66.67)	7 (28.0%)	
	No	9 (90.00)	3 (60.00)	5 (71.43)	1 (33.33)	18 (72.0%)	
**Most relatives and friends didn’t get vaccinated**							0.221
	Yes	1 (10.00)	3 (60.00)	3 (42.86)	1 (33.33)	8 (32.0%)	
	No	9 (90.00)	2 (40.00)	4 (57.14)	2 (66.67)	0 (0.00)	
**Worried about adverse reactions**							0.792
	Yes	6 (60.00)	2 (40.00)	3 (42.86)	2 (66.67)	0 (0.00)	
	No	4 (40.00)	3 (60.00)	4 (57.14)	1 (33.33)	0 (0.00)	
**What is the attitude of most patients when you recommend the influenza vaccine?**							0.052
	Interested and willing	51 (58.62)	68 (74.73)	63 (67.02)	50 (53.76)	232 (63.6%)	
	Uninterested, deem it unnecessary	5 (5.75)	7 (7.69)	6 (6.38)	10 (10.75)	28 (7.7%)	
	Indifferent, neutral to vaccination	31 (35.63)	16 (17.58)	25 (26.60)	33 (35.48)	105 (28.8%)	
**Does your institution conduct health education or training for medical staff?**							0.381
	Yes	97 (100.00)	96 (100.00)	101 (100.00)	95 (98.96)	389 (99.7%)	
	No	0 (0.00)	0 (0.00)	0 (0.00)	1 (1.04)	1 (0.3%)	
**Does your institution conduct health education or training for patients?**							0.104
	Yes	97 (100.00)	96 (100.00)	101 (100.00)	94 (97.92)	388 (99.5%)	
	No	0 (0.00)	0 (0.00)	0 (0.00)	2 (2.08)	2 (0.5%)	
**Do you provide health education or training for patients in your daily work?**							0.792
	Yes	95 (97.94)	94 (97.92)	98 (97.03)	92 (95.83)	379 (97.2%)	
	No	2 (2.06)	2 (2.08)	3 (2.97)	4 (4.17)	11 (2.8%)	
**What is the biggest difficulty or concern you or your family/friends have encountered during influenza diagnosis and treatment?**							0.185
	Inability to obtain a clear diagnosis	31 (31.96)	34 (35.42)	23 (22.77)	31 (32.29)	119 (30.5%)	
	Anti-influenza drugs are too expensive	10 (10.31)	14 (14.58)	21 (20.79)	18 (18.75)	63 (16.2%)	
	Anti-influenza drugs are difficult to access	5 (5.15)	10 (10.42)	9 (8.91)	7 (7.29)	31 (7.9%)	
	Prone to cause complications	31 (31.96)	31 (32.29)	30 (29.70)	30 (31.25)	122 (31.3%)	
	Treatment costs are too high	10 (10.31)	4 (4.17)	13 (12.87)	5 (5.21)	32 (8.2%)	
	Other	10 (10.31)	3 (3.12)	5 (4.95)	5 (5.21)	23 (5.9%)	

Note: ^#^ This is a multi-choice question.

## Data Availability

The datasets generated and/or analyzed during the current study are not publicly available due to restrictions imposed by the ethical approval and institutional data sharing policies, which protect participant confidentiality. De-identified data may be made available from the corresponding author on reasonable request, subject to review by the ethics committee.

## Supplementary Materials

The following supporting information can be downloaded at: https://www.mdpi.com/article/10.3390/vaccines14020166/s1. Table S1: Summary of univariate logistic analysis of influencing factors regarding “whether to get vaccinated against flu” among 390 HCWs in 4 provinces of China, 2025; Table S2: Summary of multivariate logistic analysis of influencing factors regarding “whether to get vaccinated against flu” among 390 HCWs in 4 provinces of China, 2025.

## Abbreviations

The following abbreviations are used in this manuscript:

HCWs	Healthcare workers.
KAP	Knowledge, attitudes, and practices.
WHO	World Health Organization.
CNIC	Chinese National Influenza Center.
CDC	Center for Disease Control and Prevention.
ILI	Influenza-like illness.
