# Supplementary material for: The Discrepancy Between Influenza Vaccine Recommendation and Uptake Among Healthcare Workers in China: A Multicenter Cross-Sectional Study
Source: Vaccines (Basel). 2026 Feb 11;14(2):166. doi: 10.3390/vaccines14020166 (PMC12945061; doi:10.3390/vaccines14020166)
Supplement: Supplementary file 1 [file vaccines-14-00166-s001.zip › vaccines-4113563-supplementary.pdf]

Process of sites selection and participants recruitment

Study sites were selected through a structured, multi-stage process considering geographical distribution (Northern vs. Southern China) and economic development levels (Eastern, Central, and Western regions) by CNIC. Leveraging established collaborative foundations, we initially consulted with leaders in charge of influenza control from several provincial Centers for Disease Control and Prevention (CDCs) to assess their interest in participation. Following these expressions of interest, candidate provinces submitted formal project proposals, which underwent a rigorous expert peer-review process. Based on these evaluations, Shanghai, Shandong, Chongqing, and Hubei were selected as the four study sites to ensure optimal regional and socioeconomic representation.

The selection process of hospitals and HCWs was carried out by provincial CDCs under the instruction of CNIC.

As stated in the manuscript, 4 cities/districts were selected into the survey in each province/municipality on consideration of regional and economic representativeness. Taking Chongqing as an example, four distinct areas—Yuzhong, Yongchuan, Nanchuan, and Zhongxian—were strategically selected as study sites. This combination effectively captures the municipality's unique urban-rural duality and socioeconomic gradient. Yuzhong District, as the highly urbanized core, represents high-density populations with abundant medical resources, whereas Zhongxian provides insights into rural dynamics and the healthcare-seeking behaviors of residents in agricultural settings. Meanwhile, Yongchuan and Nanchuan serve as representative regional centers with intermediate economic development. Consequently, this process of selection provides a comprehensive view of influenza-related knowledge, attitudes, and practices (KAP) across the diverse demographic strata of Southwest China. A similar selection logic was conducted in Shanghai, Shandong and Hubei.

Then, one primary, one secondary, and on tertiary hospitals were recruited in each of the selected city/district to ensure the cross-sectional representativeness of the local health system, on considering of structural disparity in educational background, health literacy, and clinical practices among HCWs from different levels of hospitals. The selection process was primarily based on the participation interest of hospitals and its collaboration foundation regarding influenza control and prevention with local CDC. Thus, 48 hospitals from 16 cities/districts out of 4 provinces/municipalities were determined.

Last, 8 HCWs who were directly engaged in treatment, specimen testing or vaccination regarding influenza from each hospital were recruited after informed

consent. The recruitment process was primarily based on the voluntary participation of healthcare workers, while ensuring a balanced representation across three major occupational categories: clinicians, nurses, and allied clinical technicians. Consequently, about 384 (n = 48 per site across 8 sites) HCWs were recruited as participants.

Table S1 Summary of univariate logistic analysis of influencing factors regarding "whether to get vaccinated against flu" among 390 HCWs in 4 provinces of China, 2025

		Annual vs Never			Occasional vs Never	
**Variable**	**Beta**	**OR (95% CI)**	***P***	**Beta**	**OR (95% CI)**	***P***
**Province/municipalities**						
Shanghai (Ref.)						
Shandong	1.3614	3.90 (1.61-9.45)	0.0026	0.2202	1.25 (0.58-2.70)	0.5758
Hubei	-0.379	0.68 (0.26-1.84)	0.4516	0.279	1.32 (0.67-2.63)	0.4256
Chongqing	0.0327	1.03 (0.45-2.38)	0.9388	-0.6771	0.51 (0.26-0.99)	0.0471
**Hospital level**						
Primary (Ref.)						
Secondary	-0.6931	0.50 (0.24-1.06)	0.0715	0.2199	1.25 (0.67-2.30)	0.4836
Tertiary	-0.3348	0.72 (0.36-1.43)	0.3444	0.1214	1.13 (0.62-2.07)	0.694
**Position**						
Doctor (Ref.)						
Nurse	1.197	3.31 (1.68-6.51)	<0.001	0.7696	2.16 (1.19-3.92)	0.0115
Clinical technician	-1.3552	0.26 (0.05-1.22)	0.0879	-0.8047	0.45 (0.18-1.11)	0.0829
**Work experience (years)**						
0~ (Ref.)						
10~	0.052	1.05 (0.52-2.14)	0.8858	0.6592	1.93 (1.06-3.53)	0.0322
20～	0.3323	1.39 (0.56-3.49)	0.4784	1.1565	3.18 (1.47-6.86)	0.0032
30～	0.6311	1.88 (0.69-5.09)	0.2147	-0.0339	0.97 (0.36-2.60)	0.9464

**Gender**							
	Male (Ref.)						
	Female	1.2379	3.45 (1.63-7.28)	0.0012	0.3883	1.47 (0.87-2.50)	0.1484
**Age group（years)**							
	20 ~ (Ref.)						
	30 ~	-0.1425	0.87 (0.32-2.39)	0.7826	0.0161	1.02 (0.43-2.41)	0.9707
	40 ~	0.2435	1.28 (0.45-3.60)	0.6456	0.7013	2.02 (0.83-4.87)	0.1194
	50 ~	0.3285	1.39 (0.43-4.51)	0.5846	0.0183	1.02 (0.36-2.90)	0.9726
**Ethnicity**							
	Han Chinese (Ref.)						
	Other ethnic groups				0.7605	2.14 (0.25-18.58)	0.4904
**Marital status**							
	Single (Ref.)						
	Married	-0.1712	0.84 (0.32-2.19)	0.7253	-0.2322	0.79 (0.36-1.77)	0.5708
	Divorced	-0.1066	0.90 (0.10-7.77)	0.9228	0.0366	1.04 (0.18-6.09)	0.9677
	Widowed	7.9306	2781.15 (0.00-)	0.8866	-3.9729	0.02 (0.00-)	0.9871
**Health status**							
	Good (Ref.)						
	Moderate	-0.5233	0.59 (0.21-1.71)	0.3325	-0.0765	0.93 (0.42-2.04)	0.8497
	Poor	-2.3959	0.09 (0.00-∞)	0.9812	5.9006	365.24 (0.00-∞)	0.8414
**Education background**							

	High School / Vocational School (Ref.)						
	College / University	7.0995	1211.38 (0.00-∞)	0.7642	0.8881	2.43 (0.34-17.63)	0.3796
	Master's Degree or Higher	6.6293	756.96 (0.00-∞)	0.7794	0.7659	2.15 (0.28-16.29)	0.4584
**Monthly income per capita (yuan)**							
	0~ (Ref.)						
	5000~	0.1019	1.11 (0.56-2.19)	0.7696	0.3367	1.40 (0.79-2.49)	0.251
	10000~	0.6446	1.91 (0.69-5.23)	0.2112	0.9418	2.56 (1.07-6.16)	0.0353
	20000~	-0.4006	0.67 (0.18-2.52)	0.5534	-0.2828	0.75 (0.26-2.21)	0.606
**Score grade**							
	<=70 (Ref.)						
	80 /90	-0.0645	0.94 (0.38-2.30)	0.8878	0.0646	1.07 (0.50-2.28)	0.8676
	100	0.1586	1.17 (0.45-3.07)	0.7469	0.1362	1.15 (0.50-2.63)	0.748
	>110	-0.3328	0.72 (0.30-1.71)	0.4534	-0.1677	0.85 (0.41-1.75)	0.6521

Table S2 Summary of multivariate logistic analysis of influencing factors regarding "whether to get vaccinated against flu" among 390 HCWs in 4 provinces of China, 2025

Variable		A. Annual vs Never			B. Occasional vs Never	
	Beta (SE)	OR (95% CI)	*P*	Beta (SE)	OR (95% CI)	*P*
**Hospital level**						
Primary (Ref.)						
Secondary	-0.697 (0.436)	0.50 (0.21 - 1.17)	0.110	0.144 (0.360)	1.16 (0.57 - 2.34)	0.688
Tertiary	-0.366 (0.443)	0.69 (0.29 - 1.65)	0.408	0.255 (0.380)	1.29 (0.61 - 2.72)	0.502
**Position**						
Doctor (Ref.)						
Clinical technician	-1.713 (0.842)	0.18 (0.03 - 0.94)	0.042	-1.024 (0.547)	0.36 (0.12 - 1.05)	0.061
Nurse	1.133 (0.452)	3.11 (1.28 - 7.53)	0.012	0.889 (0.396)	2.43 (1.12 - 5.29)	0.025
**Work experience (years)**						
0~ (Ref.)						
10~	-0.296 (0.538)	0.74 (0.26 - 2.13)	0.582	0.274 (0.448)	1.31 (0.55 - 3.17)	0.542
20 ~	-0.673 (0.764)	0.51 (0.11 - 2.28)	0.379	0.270 (0.634)	1.31 (0.38 - 4.54)	0.670
30 ~	-0.287 (1.092)	0.75 (0.09 - 6.38)	0.793	-0.874 (0.964)	0.42 (0.06 - 2.76)	0.365
**Gender**						
Male (Ref.)						
Female	1.126 (0.450)	3.08 (1.28 - 7.44)	0.012	0.263 (0.332)	1.30 (0.68 - 2.49)	0.427
**Age group**（years）						
20 ~ (Ref.)						
30 ~	0.124 (0.709)	1.13 (0.28 - 4.54)	0.861	0.188 (0.604)	1.21 (0.37 - 3.94)	0.755
40 ~	1.049 (0.869)	2.85 (0.52 - 15.67)	0.227	1.001 (0.733)	2.72 (0.65 - 11.46)	0.172

	50 ~	1.079 (1.195)	2.94 (0.28 - 30.63)	0.367	0.898 (1.010)	2.45 (0.34 - 17.76)	0.374
**Marital status**							
	Single (Ref.)						
	Divorced/Widowed	0.298 (1.262)	1.35 (0.11 - 15.97)	0.814	-0.803 (1.137)	0.45 (0.05 - 4.16)	0.480
	Married	-0.696 (0.675)	0.50 (0.13 - 1.87)	0.302	-0.934 (0.560)	0.39 (0.13 - 1.18)	0.096
**Health status**							
	Good (Ref.)						
	Moderate/Poor	-1.075 (0.607)	0.34 (0.10 - 1.12)	0.076	-0.498 (0.481)	0.61 (0.24 - 1.56)	0.301
**Education background**							
	College or below (Ref.)						
	Postgraduate or higher	0.020 (0.515)	1.02 (0.37 - 2.80)	0.969	-0.014 (0.399)	0.99 (0.45 - 2.16)	0.973
**Monthly income per capita (yuan)**							
	0~(Ref.)						
	5000~	0.256 (0.405)	1.29 (0.58 - 2.86)	0.527	0.491 (0.337)	1.63 (0.84 - 3.16)	0.145
	10000~	0.820 (0.587)	2.27 (0.72 - 7.17)	0.162	1.140 (0.499)	3.13 (1.17 - 8.32)	0.022
	20000~	-0.736 (0.761)	0.48 (0.11 - 2.13)	0.333	-0.622 (0.612)	0.54 (0.16 - 1.78)	0.309
**Score grade**							
	<=70 (Ref.)						
	80 /90	-0.054 (0.532)	0.95 (0.33 - 2.69)	0.919	-0.103 (0.435)	0.90 (0.38 - 2.12)	0.812
	100	-0.065 (0.567)	0.94 (0.31 - 2.85)	0.908	-0.259 (0.470)	0.77 (0.31 - 1.94)	0.581
	>110	-0.457 (0.514)	0.63 (0.23 - 1.74)	0.374	-0.464 (0.422)	0.63 (0.28 - 1.44)	0.271
